# Genomic Insights and Functional Analysis Reveal Plant Growth Promotion Traits of *Paenibacillus mucilaginosus* G78

**DOI:** 10.3390/genes14020392

**Published:** 2023-02-02

**Authors:** Dan Wang, Verena Poinsot, Wangxi Li, Yusheng Lu, Chong Liu, Yaying Li, Kaizhi Xie, Lili Sun, Chaohong Shi, Huanlong Peng, Wanling Li, Changmin Zhou, Wenjie Gu

**Affiliations:** 1 Institute of Agricultural Resources and Environment, Guangdong Academy of Agricultural Sciences, Key Laboratory of Plant Nutrition and Fertilizer in South Region, Ministry of Agriculture, Guangdong Key Laboratory of Nutrient Cycling and Farmland Conservation, Guangdong Engineering Research Center of Soil Microbes and Cultivated Land Conservation, Guangzhou 510640, China; 2 Université Toulouse III Paul Sabatier, Inserm, I2MC, Avenue Du Professeur Jean Poulhès, BP 84225, CEDEX 4, 31432 Toulouse, France; 3 Maoming Branch, Guangdong Laboratory for Lingnan Modern Agriculture, Maoming 525000, China

**Keywords:** *Paenibacillus mucilaginosus*, IAA, phosphate solubilization, Pel polysaccharide, genome analysis

## Abstract

*Paenibacillus mucilaginosus* has widely been reported as a plant growth-promoting rhizobacteria (PGPR). However, the important genomic insights into plant growth promotion in this species remain undescribed. In this study, the genome of *P. mucilaginosus* G78 was sequenced using Illumina NovaSeq PE150. It contains 8,576,872 bp with a GC content of 58.5%, and was taxonomically characterized. Additionally, a total of 7337 genes with 143 tRNAs, 41 rRNAs, and 5 ncRNAs were identified. This strain can prohibit the growth of the plant pathogen, but also has the capability to form biofilm, solubilize phosphate, and produce IAA. Twenty-six gene clusters encoding secondary metabolites were identified, and the genotypic characterization indirectly proved its resistant ability to ampicillin, bacitracin, polymyxin and chloramphenicol. The putative exopolysaccharide biosynthesis and biofilm formation gene clusters were explored. According to the genetic features, the potential monosaccharides of its exopolysaccharides for *P. mucilaginosus* G78 may include glucose, mannose, galactose, fucose, that can probably be acetylated and pyruvated. Conservation of the *pelADEFG* compared with other 40 *Paenibacillus* species suggests that Pel may be specific biofilm matrix component in *P. mucilaginosus*. Several genes relevant to plant growth-promoting traits, i.e., IAA production and phosphate solubilization are well conserved compared with other 40 other *Paenibacillus* strains. The current study can benefit for understanding the plant growth-promoting traits of *P. mucilaginosus* as well as its potential application in agriculture as PGPR.

## 1. Introduction

As the world’s population is expected to exceed 9 billion over the next thirty years, an important question to address is how to meet the increasing demands for food [1,2,3]. The application of chemical fertilizers surely helped in increasing crop yields in the last fifty years, but their intensive and continuous use has brought about a lot of environmental problems, such as diverse pollutions and contamination of ecosystems but also soil quality and biodiversity reduction [1]. Plant growth-promoting rhizobacteria (PGPR) are not only able to increase the crop production, but also have the ability to help plants to resist to biotic or abiotic stresses. Thus, the use of PGPR to substitute for part the chemical fertilizers has been considered as an eco-friendly way [4,5].

Most strains from *Paenibacillus* genus isolated from soil promote plant growth by producing indole-3-acetic acid (IAA) and other auxin phytohormones. Such bacteria can solubilize inaccessible phophorous into forms that can be taken up by plant roots, and some strains can even fix atmospheric nitrogen [6]. 

*P. mucilaginosus* was first phylogenetically characterized as *Bacillus mucilaginosus* in 1967, and it was reclassified to the genus *Paenibacillus* in 2010 [7,8]. It is widely distributed in the soil or rhizosphere and produces high yield of exopolysaccharides [9,10,11,12,13]. The strains from this species can promote the growth of green gram, trifoliate orange, maize and apple seedling, and it has thus been described as an efficient PGPR [14,15,16,17,18,19]. 

As many genes are silenced under laboratory conditions, the whole-genome sequencing (WGS) and bioinformatics tools could help biologists investigate more functions and products of PGPR. With the development of genomic era, the next generation sequencing technology makes DNA sequencing faster and cheaper than the first-generation method [20]. Thus, more and more ecologically important metabolites for PGPR have been discovered by the extensive genomic studies [21]. Until now, the genomes of three strains (KNP414, 3016 and K02) from *P. mucilaginosus* have been sequenced [22,23,24]. However, it is necessary to provide detailed genome-level descriptions of essential features in *P. mucilaginosus*, including phosphate solubilization, plant hormone production, biofilm formation, and exopolysaccharide biosynthesis.

In this study, we sequenced the genome of a strain *P. mucilaginosus* G78, and we annotated the genes related to the ability of solubilizing the phosphate, releasing IAA, producing exopolysaccharides, and forming the biofilm. We also compared genomic regions implicated in association with plant hosts among 40 other strains from *Paenibacillus* genus. This study aimed to provide a foundation for the genetic studies and functions of *P. mucilaginosus* species and explore the potential ability of plant growth promotion of *Paenibacillus* genus at the genomic level. 

## 2. Materials and Methods

### 2.1. Measurement of IAA Production and Phosphate Solubilization

For the measurement of IAA production, *P. mucilaginosus* G78 was grown in modified ACCC5 medium supplemented with 100 μg/mL Trp (IAA precursor). The modified ACCC5 medium contained sucrose 10 g/L, yeast extracts 0.5 g/L, K_2_HPO_4_·3H_2_O 0.5 g/L, NaCl 0.2 g/L, MgSO_4_·7H_2_O 0.2 g/L, CaCO_3_ 1 g/L, at pH 7.2 [22]. The production of IAA was measured by using colorimetric assay, and the modified ACCC5 medium with Trp was used as negative control [25]. For the determination of phosphate solubilization, G78 strain was inoculated into Pikovskaya’s broth containing insoluble tri-calcium phosphate (0.5%) or soybean lecithin (0.02%) and cultured for 72 h, at 30 °C. Water-soluble phosphorus in the supernatant was determined by the chlorostannous-reduced molybdophosphoric acid blue method [26].

### 2.2. Biofilm Formation Assays

The formation of biofilm was measured applying the crystal violet (CV) method following the experimental procedure as described by Shang [27]. The strain was grown overnight in modified ACCC5 medium, and the N medium was used to develop the bacterial biofilm. The N medium contained maltose 2.5 g/L, MgSO_4_·7H_2_O 0.73 g/L, K_2_HPO_4_·3H_2_O 0.4 g/L, NaCl 0.06 g/L, FeCl_3_ 0.6 mg/L, salicylic acid 10 mg/L and CaCO_3_ 1 g/L, at pH 7.2. 

### 2.3. Growth-Promoting Assay

The tomato seeds were surface sterilized by 1% (*v*/*v*) NaClO, germinated and transplanted in sterilized vermiculite moistened with Hoagland nutrient solution in Leonard jars, at 25 °C, and placed in a plant growth chamber [28]. The daylight illumination period was 12 h, and the light intensity was 1700 lx. The seedlings were inoculated with 10 mL of bacterial inoculum diluted with 10 mM sterilized MgSO_4_ solution (1 × 10^8^ cfu/mL) on the 7th, 14th, and 21st days after transplanting. The control seedlings were incorporated with the same volume of 10 mM sterilized MgSO_4_. Shoot and root lengths, fresh weight were determined at 35 days post inoculation. The root length and scanning version were acquired and analyzed by Root Analysis WINRHIZO System (Regent, CAN). Data obtained were statically analyzed using SPSS software version 25.0 (IBM Corp., Armonk, NY, USA) and were presented in tables as the means ± standard error of mean (SEM). Significant differences between treatment were compared by Independent-samples *t* test.

### 2.4. Antagonistic Activity

The antagonistic effects of *P. mucilaginosus* G78 on the fungus were detected using the dual-culture plate approach by Deng et al. [29], with some modifications. *P. mucilaginosus* G78 was inoculated and incubated on modified ACCC5 agar medium for 24 h. Fungus inhibition tests were performed by placing the agar plug with *Fusarium oxysporum* f. sp. *Momordicae* or *F. oxysporum* f. sp. *Cucumerinum* in the center of PDA medium, and three agar plugs with *P. mucilaginosus* were placed 2.5 cm from the center. The agar plugs with no bacteria were selected as negative control. Plates were incubated, at 28 °C, for 5 days and checked for inhibition.

### 2.5. Antibiotic Susceptibility Tests

Bacteria was cultured for 24 h, centrifuged, resuspended and diluted 10^2^ times with the modified ACCC5 medium, and then spread onto modified ACCC5 agar medium containing different antibiotics. Bacteria spread onto the medium without any antibiotics was used as a control [30]. The antibiotics used in this study included ampicillin, bacitracin, polymyxin, chloramphenicol, vancomycin, tetracycline, streptomycin and getamicin, with 1 mg/L, 5 mg/L, 10 mg/L, 50 mg/L, 100 mg/L and 150 mg/L, respectively. Plates were incubated, at 28 °C, and checked for inhibition.

### 2.6. Genome Sequencing and Analysis

The genomic DNA was extracted using a Qiagen Genomic-tip kit and following a modified manufacturer’s protocol as previously described [31]. Sequencing libraries were generated using NEBNext^®^ UltraTM DNA Library Prep Kit for Illumina (Lincoln, NE, USA) following manufacturer’s recommendations, and index codes were added to attribute sequences to the sample. The whole genome of *P. mucilaginosus* G78 was sequenced using Illumina NovaSeq PE150 at the Beijing Novogene Bioinformatics Technology Co., Ltd. (Beijing, China). The predicted CDSs were annotated from NR (NCBI non-redundant protein sequences; Version 202210, Swiss-Prot (A manually annotated and reviewed protein sequence database; Version 202210), Pfam (Protein family; Version Pfam v35.0), GO (Gene Ontology; Version 20220915), COG (Clusters of Orthologous Groups of proteins; Version 202006), and KEGG (Kyoto Encyclopedia of Genes and Genomes; Version 202210) database using sequence alignment tools such as RPS-BLAST, Diamond and HMMER. Briefly, each set of query proteins were aligned with the databases, and annotations of best-matched subjects (e-value < 10^−5^) were obtained for gene annotation. Secondary metabolites synthesis clusters were identified using antiSMASH (Version 5.1.2). Antibiotic resistance genes were predicted using CARD (Comprehensive Antibiotic Resistance Database, version 1.1.3). The genomic analyses were also performed using the online platform of Majorbio Cloud Platform (http://cloud.majorbio.com accessed on 1 October 2022) from Shanghai Majorbio Bio-pharm. The GenBank accession number of the sequence for *P. mucilaginosus* G78 is JAKQYK000000000.

### 2.7. Comparative Genomic and Phylogenetic Analysis

The core-orthologs from 41 strains were detected by PGAP pipeline-based protein similarity method [32]. The core-orthologs were clustered with at least 50% similarity for protein sequence to each other and 50% overlap with the longest sequence. The total genes within 41 genomes weres defined as the pan genome, and the shared genes among 41 strains was defined as their core genome [26]. Multiple alignment of amino acid sequences was carried out by using ClustalW (version 2.1) [33]. Conserved blocks from multiple alignments of test proteins were selected by using Gblocks [34]. Phylogenetic trees were inferred with 309 sing-copy core genes shared by 41 taxa. Maximum Likelihood (ML) method were inferred with PhyML (version 3.0) using the LG model with 1000 bootstrap replicates to construct the phylogenetic trees [35].

## 3. Results

### 3.1. Assessment of Plant Growth-Promoting Traits

**Figure 1:**
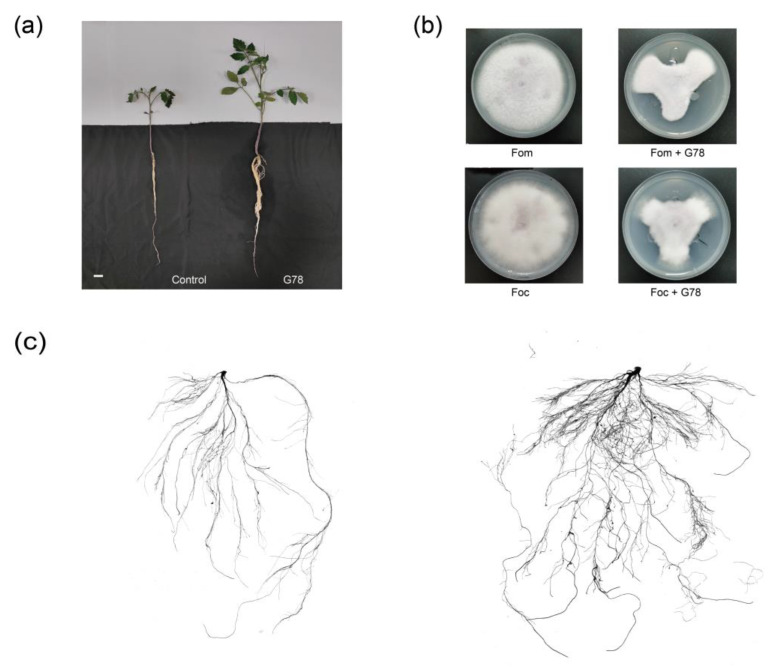
The plant growth promotion ability and antagonistic activity against pathogenic fungi through the dual-culture test of *P. mucilaginosus* G78 strain. (**a**) The effects of G78 strain on the growth of tomato plant. Bar, 2 cm. (**b**) The growth of *F. oxysporum* f. sp. *momordicae* and *F. oxysporum* f. sp. *cubense* with or without G78. Fom, *F. oxysporum* f. sp. *momordicae*; Foc, *F. oxysporum* f. sp. *cubense*; Fom+G78 or Foc+G78, F. oxysporum f. sp. momordicae or *F. oxysporum* f. sp. *cubense* with G78. (**c**) The root scanning image of tomato plants. G78, the treatment which was inoculated by G78; control, the uninoculated treatment.

Our results indicated significant effect of *P. mucilaginosus* G78 having the ability to form biofilm, solubilizing the inorganic and organic phosphate, and produce IAA (Table 1). Furthermore, the plant height and fresh weight determined after 35 days of inoculation of *P. mucilaginosus* G78 showed significant differences (*p* < 0.05), as presented in Table 2. The inoculation treatment improved the plant height and fresh weight of the tomato plant. The plant height and fresh weight of G78-treated tomato plants increased by 44.1% and 90.0% compared to the control plant, respectively (Table 2, Figure 1a,c), indicating the growth-promoting effect of *P. mucilaginosus* G78 inoculation.

### 3.2. Genomic Features

**Figure 2:**
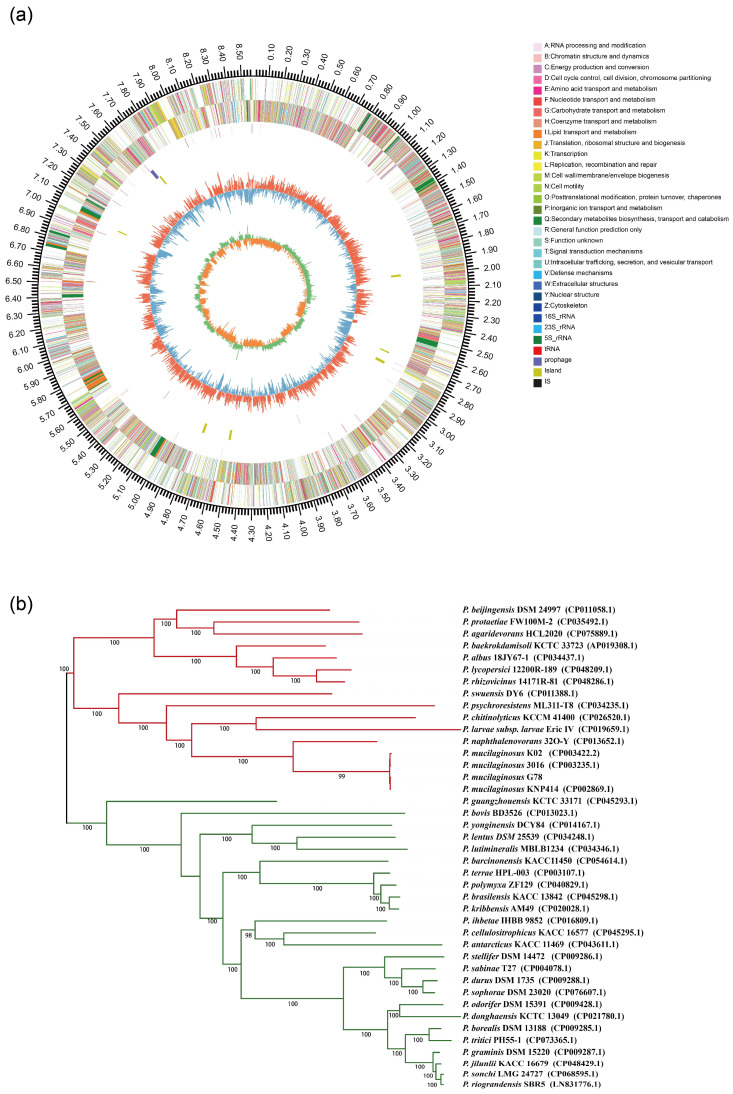
Genomic features of *P. mucilaginosus* G78 and phylogenetic relationship of 41 *Paenibacillus* strains. (**a**) Genome map of *P. mucilaginosus* G78. Circles (from outside to inside) as follows: (1) scale marks (unit, Mb), (2) protein-coding sequences on the forward strand colored by COG category, (3) protein-coding sequences on the reverse strand (same color scheme as the second circle), (4) rRNA genes, (5) tRNA genes, (6) GC content (deviation from average), and (7) positive (green) and negative (orange) GC skew. (**b**) ML phylogenetic tree was constructed using based on 309 single-copy core proteins shared by 41 genomes.

After assembly, the draft genome size of the *P. mucilaginosus* G78 was 8,576,872 bp with a GC content of 58.5% and 77 scaffolds with the N50 of 250,045 bp. The mean scaffold size was 111,388 bp and the longest scaffold was 778,093 bp. Additionally, a total of 7337 genes with 143 tRNAs, 41 rRNAs, and 5 ncRNAs were identified. The predicted genes included 2274 genes involved in metabolism, 753 genes involved in environmental information processing, and 253 genes in cellular processes. COG function classification showed that 904 genes are involved in carbohydrate transport and metabolism, 701 genes involved in the transcription, 651 genes involved in general function, and 497 genes involved in signal transduction. A total of 423 carbohydrate-active enzyme-encoding genes were identified in G78, including glycosyl hydrolysis (GHs, 58.4%), glycosyl transferases (GTs, 11.8%), carbohydrate esterases (CEs, 31.3%), carbohydrate-binding modules (CBMs, 1.2%), polysaccharide lyases (PLs, 5.7%), and auxiliary activities (AAs, 5%). The circular genome visualization for the *P. mucilaginosus* G78 was produced by the circular viewer, as shown in Figure 2a.

### 3.3. Phylogenetic Tree and Comparative Genomic Analysis

A phylogenetic tree based on single-copy core genes was reconstructed using the whole genome sequence (Figure 2b). The information about *Paenibacillus* strains was shown in Table S1. It was inferred among the 41 *Paenibacillus* strains that the G78 strain was very closely related to the *P. mucilaginosus* strain KNP414 and to two other *P. mucilaginosus* strains: K02 and 3016. The ANI value between *P. mucilaginosus* G78 and KNP414 equals 99.9%, 98.9% for strain 3016 and 98.49% for K02. It also indicated that the *P. mucilaginosus* strains grouped closely to *P. naphthalenovorans* strain 32O-Y.

To visualize the similarity of encoded proteins, the whole-genome alignments of protein coding sequences were conducted for 41 *Paenibacillus* species strains. Average amino acid identities were calculated using the pair-wise orthologous sets of CDSs. Only 0.13% of the total 234,857 putative protein-coding genes were identified as core genes, which suggests that genetic differentiation and horizontal gene acquisition from other taxa are high. G78 contained a total of 229 strain specific genes, while *P. mucilaginosus* strain KNP414 has 728 strain specific CDS.

### 3.4. Secondary Metabolites Production and Antimicrobial Resistance Genes

As shown by the dual-cultural plate, *P. mucilaginosus* G78 exhibits prohibition of the growth of the plant pathogen, *F. oxysporum* f. sp. *momordicae* and *F. oxysporum* f. sp. *cubense* (Figure 1b) following incubation for 5 d. Such findings demonstrated the capability of G78 strain to inhibit the growth of *F. oxysporum* f. sp. *momordicae* and *F. oxysporum* f. sp. *cubense*, with the inhibition rate of 51.2% and 47.3%, respectively. In *P. mucilaginosus* G78, the genome analysis identified several gene clusters that encode secondary metabolites. The putative natural products include terpene, siderophore, ladderane, flaviolins, polyketides, and NRPS. The NRPS contains some proposed peptide antibiotics, such as icosalide, paenibacterin, tridecaptin, locillomycin. The representative gene clusters encoding putative secondary metabolites were summarized in Table 3. G78 can grow under ampicillin, bacitracin, and polymyxin at a low concentration level, suggesting it contains antimicrobial resistance-related genes (Table 4). These were predicted based on the CARD database (Table 5). G78 was found to contain 429 genes related to the resistance to different antibiotics (Table S2).

### 3.5. EPS Synthesis Genes

**Figure 3:**
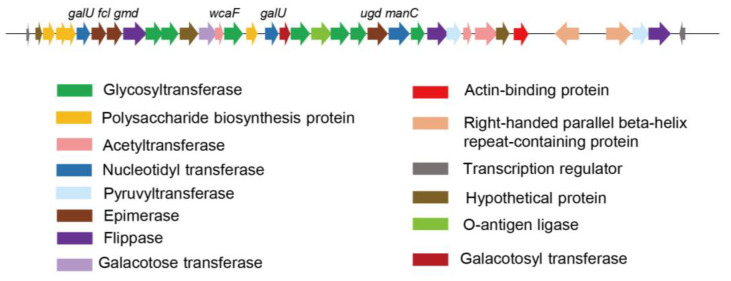
Predicted exopolysaccharide gene cluster for *P. mucilaginosus* G78. The predicted functions of each color-coded ORF are indicated at the lower bottom panel.

Exopolysaccharides (EPS) play key structural and functional roles in *P. mucilaginosus*, and were reported to protect the bacteria against the host defense during the plant–microbe interaction. We found an EPS gene cluster in G78 strain, mainly comprising 35 putative genes on a ~39.1 kb DNA fragment, which includes glycosyl transferases, polymerases, enzymes involved in the synthesis of nucleotide precursors and enzymes responsible for sugar modification or the addition of sugar substituents (Table 6, Figure 3). We further blast the putative EPS biosynthetic gene cluster among the sequenced strains from this species, and found *P. mucilaginosus* KNP414 and K02 have very similar gene structures with strain G78, while strain 3016 showed some of the truncated and non-homologous sequences (Figure S1). 

### 3.6. Biofilm Formation Genes

**Figure 4:**
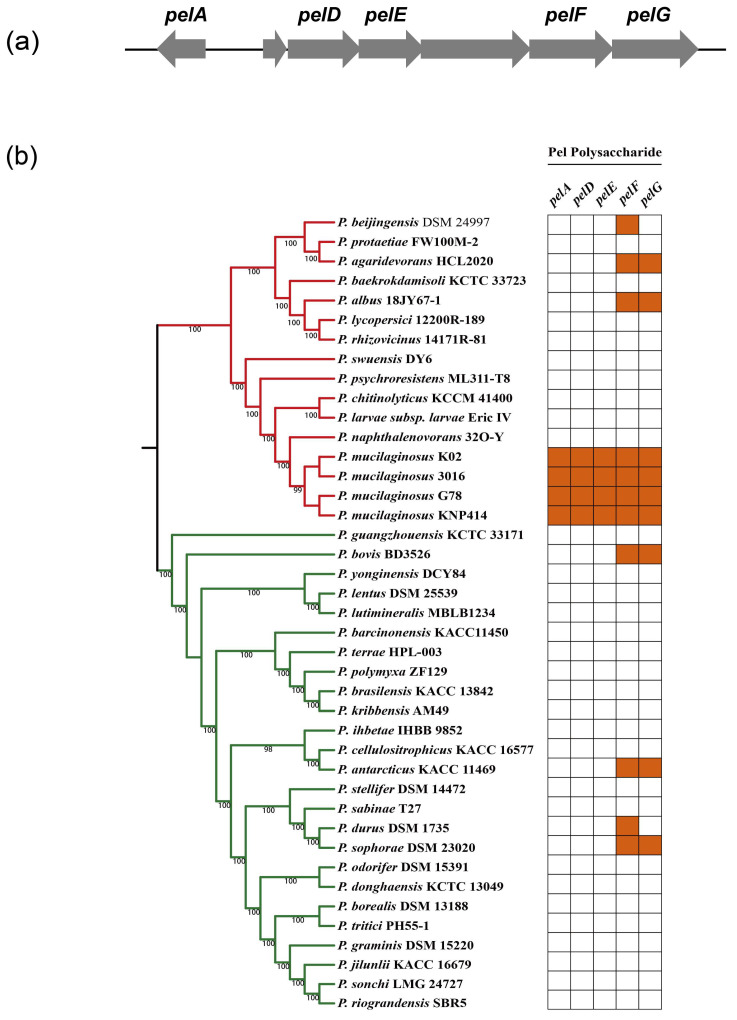
*pel*-like operon of *P. mucilaginosus* G78 and genes involved in Pel-polysaccharide of 41 *Paenibacillus* strains. (**a**) *pel*-like operon architectures of *P. mucilaginosus* G78. Arrows are used to denote open reading frames, with the direction of each arrow indicating the direction of transcription. (**b**) *pelAEDFG* genes of 41 *Paenibacillus* strains. Colored box represents the presence of a gene within a genome and white box indicates absence of a gene.

The key genes involved in the formation of biofilm were investigated using the KAAS database, and 28 genes were explored, including metabolic pathway regulators, diguanylate or adenylate cyclase, matrix protein-coding genes, and putative matrix polysaccharide synthesis genes (Table 7). It was shown that *pel*-like operon encoded the biofilm polysaccharide in *Bacillus cereus* [36]. We explored the *pel*-like genes among 41 *Paenibacillus* strains, and found that *P. mucilaginosus* strains had more Pel polysaccharide biosynthetic genes, which indicated that Pel polysaccharide is not a common biofilm matrix component among the genus of *Paenibacillus* (Figure 4).

### 3.7. Plant Growth-Promoting Ability Genes

**Figure 5:**
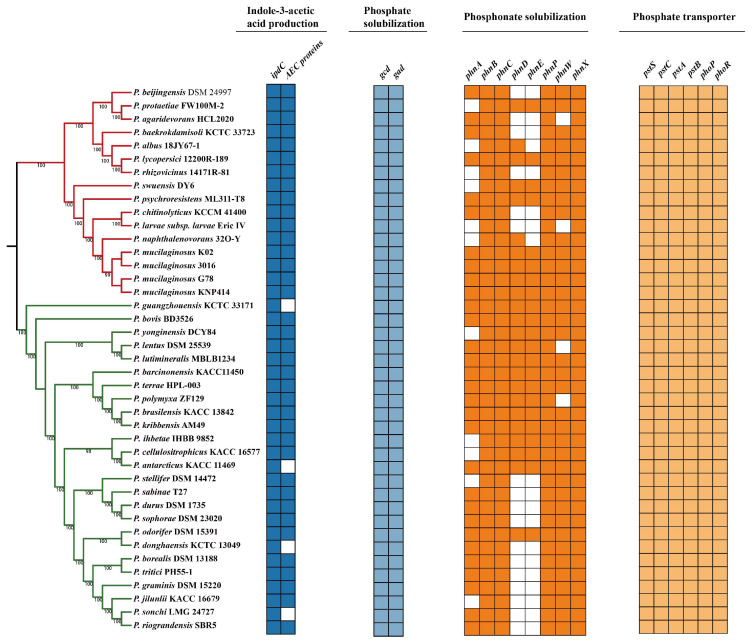
Genes involved in IAA production, organic and inorganic phosphate solubilization of 41 *Paenibacillus* strains. Colored box represents the presence of a gene within a genome and white box indicates absence of a gene.

Indole-3-acetic acid (IAA) has been reported as an important phytohormone with the capacity to control plant development, which can be produced by many rhizosphere bacteria [37]. In this study, the indolepyruvate decarboxylase (encoded by *ipdC* gene) and auxin carrier protein were identified in strain G78 (Figure 5). However, the genes encoding tryptophan monooxygenas or indole-3-acetamide hydrolase were not detected in the G78 strain. Furthermore, the gene *ipdC* exists among all tested strains, suggesting these bacteria are all capable of IAA production following the indole-3-pyruvic acid pathway, even if auxin carrier proteins are deficient in some strains.

Considering the phosphate-providing ability of strain G78, we found eight *phn* genes (*phnABCDEWXM*)and two genes encoding glucose-1-dehydragenase (*gcd*) and gluconic acid dehydrogenase (*gad*). We also identified the putative *pst* operon (*pstS*, *ptsC*, *pstA*, *pstB*) and PhoP-PhoR system in the genome of G78 (Figure 5). In addition, we screened 20 pathways in total related with the organic acid metabolic pathway, and all above can explain the phosphate solubilization and secretion ability of this strain (Table S2). Additionally, the mineral phosphate solubilizations genes (*gcd*, *gad*) and phosphate transport system are present in all 41 sequenced *Paenibacillus* strains, indicating that all these strains have the ability to promote plant growth on phosphate-limited soil.

## 4. Discussion

*P. mucilaginosus* has been widely reported as a plant growth-promoting rhizobacteria (PGPR) [14,15,16,17,18,19]. Until now, only three strains from *P. mucilaginosus* were sequenced, and the PGPR traits at the genome level has not been described in detail. In this study, we sequenced a PGPR strain, *P. mucilaginosus* G78, and explored the genes related to microbe-–plant interaction, such as secondary metabolites synthesis, exopolysaccharides biosynthesis, biofilm formation, IAA production, and phosphate-dissolving ability. The genome size of *P. mucilaginosus* G78 was 8,576,872 bp with a GC content of 58.5%, which is very similar in size with other *P. mucilaginosus* strains. As shown in Table S1, *P. mucilaginosus* showed high GC content. Moreover, it presents the second largest genome size than other 37 *Paenibacillus* strains. Focusing on the influence of several properties including biochemical, genetic flows, selection biases, and the biochemical-energetic properties shaping genome composition, it indicated a trend toward high GC content and larger genomes in free-living organisms, as a result of more complex and varied environments [38,39]. The genes related to the glycoside hydrolase family are much abundant in this strain in comparison to other *Paenibacillus*, which is consistent with their reported importance for *Paenibaicllus* survival [40].

Secondary metabolites (SM) produced by plant-associated biocontrol bacteria can directly reduce the pathogen’s ability to cause disease, induce plant defense mechanisms, or promote plant development [41]. *P. mucilaginosus* G78 showed antifungal activity against phytopathogens such as *F. oxysporum*, and has the genomic potential to produce a lot of SMs. Recent extensive bacterial genome sequencing and bioinformatic analysis showed that terpene synthases are widely distributed in bacteria [42,43]. The ability to produce or capture siderophores makes the bacteria competitive advantages to colonize plant tissues [44]. Antismash analysis showed that strain G78 has *asb* operon, which is responsible for petrobactin biosynthesis in *Bacillus anthracis* [45,46]. Kedarcidin (KED) is an aromatic enediyne that may be produced by strain G78. It was reported to be chromoprotein antitumor antibiotic and was isolated from *Streptoalloteichus* sp. ATCC 53560 but rarely reported in *Paenibacillus* or *Bacillus* genus [47,48]. Bacteriocins are ribosomally synthesized peptides (RSPs) that contain 12~50 amino acid residues which exhibited a broad spectrum of antimicrobial activity. Many of the polyketides (PKs) produced by *Bacillus* and *Paenibacillus* species have been described as bioactive natural products that had medical value and can be potentially applied in agriculture for controlling plant pathogens [49,50]. In total, there are 18 NRPS or NRPS-like metabolites proposed gene clusters in strain G78, including 10 unknown ones. The NRPS contains some proposed peptide antibiotics, such as icosalide, paenibacterin, tridecaptin, locillomycin and other new products. However, whether the metabolites mentioned above were produced still needs further determination. In this study, we demonstrated that *P. mucilaginosus* G78 could grow on the medium supplemented with ampicillin, bacitracin, polymyxin and chloramphenicol. The putative genes which have a role in the resistance to these antibiotics are listed in Table 5. The G78 strain remains susceptible to vancomycin, tetracycline and streptomycin (Table 4), although partial genes participating in these antibiotic resistances were identified by CARD analysis, indicating that the completed operon is necessary for the antibiotic resistance.

Exopolysaccharides secreted by *P. mucilaginosus* strains was reported to have strong antioxidant abilities [12] and was hypothesized to play an important role during the process of wastewater treatment [13,51]. Studying the genes responsible to EPS synthesis will be helpful to explore its potential functions and structures. Exopolysaccharide biosynthesis for bacteria usually includes the following steps: uptake of substance, nucleotide sugar precursors synthesis, assembling and polymerization, modification, and release [52]. In this study, we explored the potential EPS biosynthesis gene cluster from strain G78. It was shown that the potential monosaccharides include glucose, mannose, galactose, fucose, that can probably be acetylated and pyruvated according to its genetic features. The chemical structure of EPS from this species is strain specific, as different strains from *P. mucilaginosus* produced various EPS, consistently with the reported biosynthetic genes variations (Figure S1). The reported partial structure of the EPS from *P. mucilaginosus* SM-01 was mainly composed of β-1, 4-linked-Glc and β-1, 4-linked-Man as the backbone and branched at C-2 position of β-1, - 4-linked-Glc residue by the acetyl esters [13]. The possible structure of polysaccharide from *P. mucilaginosus* WL412 was identified as [→4)α-Glc(1 → 2)α-Man(1 → 3)β-Glc(1 → 3)α-Man(6-Ac)(1 → 3)β-Gal(1→] [53]. 

Biofilms are surface-associated microbial communities in which the cells are embedded within an extracellular matrix, and they can help the microorganisms to defend against biotic or abiotic stress, to colonize the plant host and to acquire nutrients or genetic traits [54,55,56]. The major genes for the formation of biofilm include those encoding for important biofilm transcriptional regulators, the matrix structural synthesis (matrix protein, putative matrix polysaccharide), extracellular DNA synthesis and cyclic-di-GMP metabolisms [57,58]. The genome of G78 strain contained several genes that participate in biofilm formation, including transcriptional regulators, matrix structural synthesis genes, eDNA synthesis genes and diguanylate or adenylate cyclase-encoding genes. Pel polysaccharide was reported to play an important role in the biofilm formation of *Pseudomonas aeruginosa* and *Bacillus cereus*, and their biosynthesis requires an inner membrane complex comprising of PelD, PelE, PelF, and PelG [34,59,60]. Conservation of the *pelADEFG* among 41 studied strains from the *Paenibacillus* genus suggests that Pel may not be a common biofilm matrix component in this genus except for the species of *P. mucilaginosus*. However, further investigation via a gene deletion approach is required to characterize the function of Pel polysaccharide in *P. mucilaginosus* G78.

*Paenibacillus* strains are well known for their beneficial effects of plant growth, including production of IAA and mineral solubilization [6,61,62]. The biosynthesis of indole-3-acetic acid (IAA) is often related to beneficial effects of PGPR on plant development including cell division, elongation, tropism, apical dominance, senescence, flowering, and response to stress [35,63,64,65]. In this study, *P. mucilaginosus* G78 was demonstrated to produce indole-3-acetic acid (IAA), and to promote the growth of tomato seedlings by increasing the root length, fresh weight, and height of plants. The genes encoding putative indole pyruvate decarboxylase (IpdC) and auxin efflux carrier (AEC) protein are present in the genome of *P. mucilaginosus* G78. Knocking out the *ipdC* gene in *Bacillus thuringiensis* RZ2MS9 resulted in the decreasing production of IAA and significantly reduced its ability to promote maize growth, indicating that IAA biosynthesis by this PGPR is a major mechanism to promote plant growth [66]. Xie et al. found that *ipdC* homologies are present in all analyzed *P. polymyxa* genomes, with over 96% amino acid identity between strains across 98% of the sequence [26]. We explored the *ipdC* and auxin efflux carrier protein-encoding genes among the genomes of 41 *Paenibacillus* strains, and found that *ipdC* is present in all analyzed genomes as well. In contrast, not all strains have the auxin efflux carrier protein. This could indicate that their capability of exporting IAA is not common in this genus. 

A large proportion of organic and inorganic phosphate is present in the soil, but they cannot be absorbed directly by plants because of the insoluble forms. Phosphate solubilizing bacteria has the ability to convert insoluble phosphates and to make it accessible to the plants [67,68]. It was proved that mineral phosphates solubilization is achieved through gluconic acid production and that the *phn* genes are responsible for solubilizing organic phosphate [69,70,71]. The glucose-1-dehydrogenase (*gcd*) and gluconic acid dehydrogenase (*gad*) are implicated in the production of gluconic acid [68,72,73]. The phosphate transportation is mostly related to the Pst (phosphate-specific transport) system and to the PhoP-PhoR system [24,74,75,76]. The *gcd*, *gad*, *phnABCDEPWX, pst SCAB* and *phoPR* were all present in the genome of G78, which is consistent with its ability to dissolve both organic or inorganic phosphorus compounds. All the analyzed *Paenibacillus* strains exhibit the genes for mineral phosphorus solubilization and phosphorus transport, indicating the potential application of *Paenibacillus* strains as phosphorus activator in the plant rhizosphere. In addition, although G78 strain can grow on the free-nitrogen medium, we did not find the *nif* genes present in this species (Figure S2), indicating that it could employ an unknown metabolic pathway to survive under nitrogen deficient condition, which needs to be clarified by further investigation.

## 5. Conclusions

The genome size of the *P. mucilaginosus* G78 was 8,576,872 bp with a GC content of 58.5%. Additionally, a total of 7337 genes with 143 tRNAs, 41 rRNAs, 5 ncRNAs were identified. It contained 26 gene clusters encoding secondary metabolites and 20 proteins related to the resistance to ampicillin, bacitracin, polymyxin and chloramphenicol, which is in accordance with its antagonist activity and antibiotic resistance ability. According to the genetic features, the potential monosaccharides of its exopolysaccharides for *P. mucilaginosus* G78 may include glucose, mannose, galactose, fucose, that can probably be acetylated and pyruvated. Conservation of the *pelADEFG* compared with other 40 *Paenibacillus* species suggests that Pel may be a specific biofilm matrix component in *P. mucilaginosus*. The containing genes encoding IAA production and phosphate solubilization associated with the phenotypic analysis highlighted the capability of *P. mucilaginosus* G78 strain to promote the plant growth. *P. mucilaginosus* species showed high GC content, and it presents the second largest genome size than other 37 studied *Paenibacillus* strains. Several genes associated with plant growth-promoting traits, i.e., IAA production and phosphate solubilization, are well conserved among 41 *Paenibacillus* strains, suggesting their potential uses in agriculture. 

## Figures and Tables

**Table 1:** Plant growth-promoting traits of *P. mucilaginosus* G78 strain.

	Soluble P for Inorganic Phosphate (mg/L)	Soluble P for Organic Phosphate (mg/L)	IAA Production (mg/L)	Biofilm Biomass (OD560)
G78	13.6 ± 3.1	0.48 ± 0.01	10.9 ± 0.2	0.32 ± 0.02

Notice: Values are presented as mean ± SEM of three replications.

**Table 2:** Effects of *P. mucilaginosus* G78 strain on the growth of tomato seedlings.

	Root Length * (cm Plant^−1^)	Root FW ** (g Plant^−1^)	Shoot Length ** (cm Plant^−1^)	Shoot FW ** (g Plant^−1^)
Control	435.3 ± 58.8 ^a^	0.22 ± 0.03 ^a^	16.1 ± 0.8 ^a^	0.90 ± 0.06 ^a^
G78	1001.2 ± 131.5 ^b^	0.78 ± 0.07 ^b^	23.2 ± 1.3 ^b^	1.71 ± 0.15 ^b^

Notice: *, Values are presented as mean ± SEM of three replications; **, Values are presented as mean ± SEM of five replications. Different letters within the same column indicate significant differences among treatment means according to Independent-samples *t* test (*p* < 0.01).

**Table 3:** The putative gene cluster encoding secondary metabolites in *P. mucilaginosus* G78.

Cluster ID	Type	Similar Cluster	Similarity (%)	MIBiG Accession
1	NRPS	zwittermicin A	7	BGC0001059
2	NRPS	-	-	-
3	NRPS	-	-	-
4	terpene	-	-	-
5	T3PKS	-	-	-
6	NRPS	-	-	-
7	transAT-PKS	difficidin	20	BGC0000176
8	LAP	-	-	-
9	siderophore	petrobactin	83	BGC0000942
10	bacteriocin	-	-	-
11	NRPS	locillomycin	42	BGC0001005
12	terpene	carotenoid	33	BGC0000645
13	NRPS	stigmatellin	15	BGC0000153
14	NRPS	-	-	-
15	ladderane	kedarcidin	1	BGC0000081
16	NRPS	-	-	-
17	NRPS	cyclomarin D	8	BGC0000333
18	NRPS	paenibacterin	60	BGC0000400
19	NRPS	-	-	-
20	NRPS	-	-	-
21	NRPS	tridecaptin	60	BGC0000449
22	NRPS	cystothiazole A	11	BGC0000982
23	NRPS	-	-	-
24	NRPS	-	-	-
25	NRPS	-	-	-
26	NRPS-like	icosalide A/icosalide B	100	BGC0001833

**Table 4:** Antimicrobial susceptibility test of *P. mucilaginosus* G78.

Antibiotics (μg/mL)	0	1	5	10	50	100	150
Ampicillin	+	+	+	+	+	+	-
Bacitracin	+	+	+	+	+	+	+
Polymyxin	+	+	+	+	-	-	-
Chloramphenicol	+	+	-	-	-	-	-
Vancomycin	+	-	-	-	-	-	-
Tetracycline	+	-	-	-	-	-	-
Streptomycin	+	-	-	-	-	-	-

Notice: ‘+’ represents the bacteria that can grow on the agar plates but ‘-’ represents not.

**Table 5:** The putative antibiotic resistance-related genes.

Antibiotic	ARO Name	ARO Description
ampicillin	ampH β-lactamase	ampC-like β-lactamase and penicillin-binding protein
	ampC1 β-lactamase	β-lactamase
	ampC β-lactamase	β-lactamase
	ampC1 β-lactamase	β-lactamase
	LRA-2	β-lactamase
	BcI	β-lactamase I
	SMB-1	hydrolyze a variety of β-lactams
	BUT-1	cephalosporinase and penicillinase
bacitracin		
	bcrA	ABC transporter that confers bacitracin resistance
	bacA	recycles undecaprenyl pyrophosphate that confers bacitracin resistance
	bcrB	ABC transporter that confers bacitracin resistance
	bacA	recycles undecaprenyl pyrophosphate that confers bacitracin resistance
polymyxin		
	arnA	modifies lipid A with 4-amino-4-deoxy-L-arabinose (Ara4N) that confers antimicrobial peptides resistance
	ugd	synthesis and transfer of 4-amino-4-deoxy-L-arabinose (Ara4N) to Lipid A that confers antimicrobial peptides resistance
	rosA	efflux pump/potassium antiporter system that confers resistance to cationic antimicrobial peptides
	PmrF	required for the synthesis and transfer of 4-amino-4-deoxy-L-arabinose (Ara4N) to Lipid A, which confers antimicrobial peptides resistance
chloramphenicol		
	fexA	chloramphenicol exporter
	cmlv	chloramphenicol phoshotransferase
	cmrA	chloramphenicol exporter
	cmlR	chloramphenicol resistance determinant (putative transmembrane protein)

**Table 6:** Putative exopolysaccharides production-related gene cluster in G78.

Predicted Gene Products	Length [aa]	Predicted Function	NR Description
	65	helix-turn-helix transcriptional regulator	Cro/C1-type HTH DNA-binding domain
	147	Hypothetical protein	
	254	polysaccharide biosynthesis protein	Tyrosine-protein phosphatase YwqE
	246	Polysaccharide biosynthesis protein	Chain length determinant protein
	226	Polysaccharide biosynthesis protein	Tyrosine-protein kinase YwqD
*galU*	297	Nucleotidyl transferase	UTP--glucose-1-phosphate uridylyltransferase GalU
*fcl*	318	Epimerase	GDP-L-fucose synthase
*gmd*	331	Epimerase	GDP-mannose 4,6-dehydratase
	519	Flippase	oligosaccharide flippase family protein
	378	Glycosyltransferase	Glycosyltransferase family 4 protein
	379	Glycosyltransferase	Glycosyltransferase group 1
	407	Hypothetical protein	
	393	Galactose transferase	α-galactose transferase
*wcaF*	187	acetyltransferase	colanic acid biosynthesis acetyltransferase WcaF
	412	glycosyltransferase	glycosyltransferase family 4 protein
	247	Polysaccharide biosynthesis protein	Chain length determinant protein
*galU*	297	Nucleotidyl transferase	UTP--glucose-1-phosphate uridylyl-transferase GalU
	232	sugar transferase	Galactosyl transferase
	420	glycosyltransferase	glycosyltransferase
	452	O-Antigen ligase	O-antigen ligase family protein
	413	glycosyltransferase	glycosyltransferase family 4 protein
	351	glycosyltransferase	GDP-mannose: glycolipid 4-β-D-mannosyltransferase
*ugd*	443	Epimerase	UDP-glucose/GDP-mannose dehydrogenase family protein
*manC*	460	Nucleotidyl transferase	Mannose-6-phosphate isomerase
	299	glycosyltransferase	glycosyltransferase family 2 protein
	463	Flippase	oligosaccharide flippase family protein
	325	Polysaccharide pyruvyl transferase	polysaccharide pyruvyl transferase family protein
	181	acetyltransferase	O-acetyltransferase
	500	acetyltransferase	membrane-bound O-acyltransferase
	296	Hypothetical protein	Hypothetical protein
	321	Actin-binding protein	Actin-binding protein
	528		right-handed parallel β-helix repeat-containing protein
	558		right-handed parallel β-helix repeat-containing protein
	362	pyruvyl transferase	polysaccharide pyruvyl transferase family protein
	490	flippase	oligosaccharide flippase family protein

**Table 7:** Putative biofilm formation-related genes in *P. mucilaginosus* G78.

KEGG Orthology	Genes	Protein Product
	metabolic pathway regulators	
K10914	*crp*	CRP/FNR family transcriptional regulator, cyclic AMP receptor protein
K03092	*rpoN*	RNA polymerase sigma-54 factor
K03666	*hfq*	host factor-I protein
K02398	*flgM*	negative regulator of flagellin synthesis FlgM
K03563	*csrA*	carbon storage regulator
K02405	*fliA*	RNA polymerase sigma factor for flagellar operon FliA
	diguanylate or adenylate cyclase	
K21023	*mucR*	diguanylate cyclase
K01768		adenylate cyclase
K21019	*sadC*	diguanylate cyclase
	Matrix protein-encoding genes	
K13280	*sipW*	Signal peptidase I
	Putaitve matrix polysaccharide synthesis genes	
K00640	*cysE*	serine O-acetyltransferase
K00975	*glgC*	glucose-1-phosphate adenylyl-transferase
K02777	*crr*	sugar PTS system EIIA component
K05946	*tagA*	N-acetylglucosaminyl, diphosphoundecaprenol, N-acetyl-β-D-mannosaminyl-transferase
K00688	*glgP*	glycogen phosphorylase
K01657	*trpE*	anthranilate synthase
K01791	*wecB*	UDP-N-acetylglucosamine 2-epimerase
K00703	*glgA*	starch synthase (glycosyl-transferring)
K21006	*pelA*	Glycoside-hydrolase family protein
K21009	*pelD*	NAD-dependent epimerase dehydratase
K21010	*pelE*	Polysaccharide biosynthesis protein PelE
K21011	*pelF*	Glycosyl transferase
K21012	*pelG*	Putative exopolysaccharide Exporter
	eDNA synthesis genes	
K01939	*purA*	Adenylosuccinate synthase
K01923	*purC*	Phosphoribosyl, aminoimidazole, succinocarboxamide synthase
K23269	*purL*	Phosphoribosyl, formylglycinamidine synthase subunit PurL

## Data Availability

The data presented in this study are available from the corresponding author on reasonable request.

## Supplementary Materials

The following supporting information can be downloaded at: https://www.mdpi.com/article/10.3390/genes14020392/s1, Table S1: The genome information of *Paenibacillus* strains in this study; Table S2. Putative genes related to phosphate solubilization ability in G78; Predicted exopolysaccharide gene clusters for four *P. mucilaginosus* strains (3016, K02, KNP414 and G78); Figure S1. Predicted exopolysaccharide gene clusters for four *Paenibacillus mucilaginosus* strains (3016, K02, KNP414 and G78); Figure S2. Genes involved in nitrogen fixation of 41 *Paenibacillus* strains.
