# Supplementary material for: Genomic Insights and Functional Analysis Reveal Plant Growth Promotion Traits of Paenibacillus mucilaginosus G78
Source: Genes (Basel). 2023 Feb 2;14(2):392. doi: 10.3390/genes14020392 (PMC9956331; doi:10.3390/genes14020392)
Supplement: Supplementary file 1 [file genes-14-00392-s001.zip › genes-2181233-supplementary.pdf]

Table S1. The genome information of *Paenibacillus* strains in this study.

Strains	Isolation source	Genome size (Mb)	G+C content	Protein	rRNA	tRNA	Other RNA genes	Gene number	Accession number
Paenibacillus sophorae DSM23020	Soil (South Korea)	5.87	50.2	5510	27	85	4	5724	GCA_018966525.1
Paenibacillus agaridevorans HCL2020	Rat bone (China)	9.04	51.5	6973	24	96	5	7177	GCA_018604145.1
Paenibacillus tritici PH55-1	Soil (South Korea)	7.71	51.8	6462	30	84	4	6661	GCA_018207515.1
Paenibacillus sonchi LMG24727	Soil (South Korea)	7.32	50.9	6198	13	67	5	6396	GCA_016772475.1
Paenibacillus barcinonensis KACC11450	Soil (South Korea)	6.39	47.0	5624	36	103	4	5844	GCA_013347305.1
Paenibacillus jilunlii KACC 16679	Soil (South Korea)	7.11	50.9	5992	30	86	5	6217	GCA_010450915.1
Paenibacillus rhizovicinus 14171R-81	Tomato rhizosphere (South Korea)	7.53	54.6	6561	39	125	5	6805	GCA_010365285.1
Paenibacillus lycopersici 12200R-189	Tomato rhizosphere (South Korea)	6.74	58.1	5.729	33	98	4	5926	GCA_010119935.1
Paenibacillus psychroresistens ML311-T8	Arctic soil (Norway)	8.1	41.1	7059	24	74	4	7262	GCA_009728935.1
Paenibacillus brasilensis KACC13842	Soil (South Korea)	5.64	46.1	4979	34	92	4	5376	GCA_009363115.1
Paenibacillus cellulositrophicus KACC16577	Thai soil (South Korea)	7.43	53.1	6578	27	90	4	6773	GCA_009363095.1
Paenibacillus guangzhouensis KCTC33171	Forest soil (South Korea)	6.94	47.5	6093	27	74	4	6294	GCA_009363075.1
Paenibacillus antarcticus KACC 11469	Sediment (South Korea)	5.42	40.6	4761	37	111	4	4992	GCA_008369725.1
Paenibacillus polymyxa ZF129	Potato rhizosphere soil (China)	5.82	45.3	4915	42	111	4	5133	GCA_006274405.1
Paenibacillus protaetiae FW100M-2	Protaetia brevitarsis seulensis	4.23	51.5	3698	27	91	5	3987	GCA_004135365.1
Paenibacillus chitinolyticus KCCM 41400	Chitin (Japan)	6.51	52.4	5735	34	108	4	6031	GCA_004117095.1
Paenibacillus lutimineralis MBLB1234	Bentonite (South Korea)	6.5	46.4	5600	30	104	4	5936	GCA_003991425.1
Paenibacillus albus 18JY67-1	Soil (South Korea)	6.63	50.8	5854	39	80	4	6173	GCA_003952225.1
Paenibacillus baekrokdamisoli KCTC33723	Soil (South Korea)	7.26	45.3	6235	27	74	4	6560	GCA_003945345.1
Paenibacillus lentus DSM25539	Soil (USA)	5.29	46.5	4486	24	75	4	4871	GCA_003931855.1
Paenibacillus larvae subsp. larvae Eric_IV	Honeybees (Germany)	4.38	44.2	4133	24	79	4	4720	GCA_002951935.1
Paenibacillus ihbetae IHBB 9852	Sediment (India)	6.59	52.1	5733	28	73	4	8960	GCA_002741055.1
Paenibacillus kribbensis AM49	Soil (South Korea)	5.78	46.8	4940	30	92	4	5290	GCA_002240415.1
Paenibacillus donghaensis KCTC 13049	Sea sediment (South Korea)	8.54	49.7	7372	27	94	4	7957	GCA_002192415.1
Paenibacillus bovis BD3526	yak milk (China)	5.51	47.2	4648	28	86	4	4872	GCA_001421015.2
Paenibacillus yonginensis DCY84	Humus soil (South Korea)	4.99	51.0	4344	30	84	4	4680	GCA_001685395.1
Paenibacillus swuensis DY6	Soil (South Korea)	5.01	48.9	4394	30	87	4	4595	GCA_001644605.1

Paenibacillus naphthalenovorans 32O-Y	Soil (USA)	5.2	49.7	4935	34	93	4	5238	GCA_001465255.1
Paenibacillus terrae HPL-003	Soil (South Korea)	6.08	46.8	5206	28	90	4	5684	GCA_000235585.1
Paenibacillus riograndensis SBR5	Soil (Germany)	7.92	51.0	6571	27	87	5	6892	GCA_000981585.1
Paenibacillus beijingensis DSM 24997	Jujube rhizosphere soil (China)	5.75	52.5	4979	28	89	4	5360	GCA_000961095.1
Paenibacillus odorifer DSM 15391	Wheat rhizosphere soil (France)	6.81	44.2	5863	30	87	4	6112	GCA_000758725.1
Paenibacillus graminis DSM 15220	Maize rhizosphere soil (France)	7.17	50.6	6066	30	89	4	6387	GCA_000758705.1
Paenibacillus stellifer DSM 14472	food-packaging paperboard	5.66	53.5	5046	33	87	5	5391	GCA_000758685.1
Paenibacillus borealis DSM 13188	Norway spruce forest humus	8.16	51.4	6809	30	86	5	7108	GCA_000758665.1
Paenibacillus durus DSM 1735	Marine sediment	6.06	50.8	5239	27	83	4	5545	GCA_000756615.1
Paenibacillus sabinae T27	Rhizosphere soil (China)	5.27	52.6	4750	26	82	4	5026	GCA_000612505.1
Paenibacillus mucilaginosus K02	Soil (Chiina)	8.82	58.3	7157	39	150	5	7563	GCA_000258535.2
Paenibacillus mucilaginosus 3016	Rhizosphere soil (China)	8.74	58.3	7055	42	173	6	7890	GCA_000250655.1
Paenibacillus mucilaginosus KNP414	Soil (China)	8.66	58.4	7050	39	110	5	7442	GCA_000218915.1
Paenibacillus mucilaginosus G78[*]	Soil (China)	8.58	58.5	7050	41	143	5	7338	GCA_022343625.1

*, Genomes determined in this study have been deposited at GenBank under the BioProject no. PRJNA224116. The version of the genome described in this paper is the first version.

Table S2. Putative genes related to phophate solubilization ability in G78.

KEGG ID	Function	Pathway_ID	Pathway_Description
2.7.1.40	pyruvate kinase	ko00010	Glycolysis / Gluconeogenesis
2.7.1.40	pyruvate kinase	ko00010	Glycolysis / Gluconeogenesis
2.3.3.1	citrate synthase	ko00020	Citrate cycle (TCA cycle)
2.7.1.40	pyruvate kinase	ko00230	Purine metabolism
2.7.1.40	pyruvate kinase	ko00230	Purine metabolism
2.3.3.13	2-isopropylmalate synthase	ko00290	Valine, leucine and isoleucine biosynthesis
2.3.3.13	2-isopropylmalate synthase	ko00290	Valine, leucine and isoleucine biosynthesis
2.3.3.13	2-isopropylmalate synthase	ko00290	Valine, leucine and isoleucine biosynthesis
2.7.1.71	shikimate kinase	ko00400	Phenylalanine, tyrosine and tryptophan biosynthesis
2.7.2.1	acetate kinase	ko00430	Taurine and hypotaurine metabolism
2.7.2.1	acetate kinase	ko00620	Pyruvate metabolism
2.3.3.13	2-isopropylmalate synthase	ko00620	Pyruvate metabolism
2.7.1.40	pyruvate kinase	ko00620	Pyruvate metabolism
4.1.1.31	phosphoenolpyruvate carboxylase	ko00620	Pyruvate metabolism
2.3.3.13	2-isopropylmalate synthase	ko00620	Pyruvate metabolism
2.3.3.13	2-isopropylmalate synthase	ko00620	Pyruvate metabolism
2.7.1.40	pyruvate kinase	ko00620	Pyruvate metabolism
2.3.3.1	citrate synthase	ko00630	Glyoxylate and dicarboxylate metabolism
2.7.2.1	acetate kinase	ko00640	Propanoate metabolism
2.7.2.1	acetate kinase	ko00680	Methane metabolism
4.1.1.31	phosphoenolpyruvate carboxylase	ko00680	Methane metabolism
4.1.1.31	phosphoenolpyruvate carboxylase	ko00710	Carbon fixation in photosynthetic organisms
2.7.2.1	acetate kinase	ko00720	Carbon fixation pathways in prokaryotes

4.1.1.31	phosphoenolpyruvate carboxylase	ko00720	Carbon fixation pathways in prokaryotes
2.7.1.71	shikimate kinase	ko01100	Metabolic pathways
2.7.2.1	acetate kinase	ko01100	Metabolic pathways
2.3.3.13	2-isopropylmalate synthase	ko01100	Metabolic pathways
2.7.1.40	pyruvate kinase	ko01100	Metabolic pathways
4.1.1.31	phosphoenolpyruvate carboxylase	ko01100	Metabolic pathways
2.3.3.13	2-isopropylmalate synthase	ko01100	Metabolic pathways
2.3.3.13	2-isopropylmalate synthase	ko01100	Metabolic pathways
2.7.1.40	pyruvate kinase	ko01100	Metabolic pathways
2.3.3.1	citrate synthase	ko01100	Metabolic pathways
2.7.1.71	shikimate kinase	ko01110	Biosynthesis of secondary metabolites
2.3.3.13	2-isopropylmalate synthase	ko01110	Biosynthesis of secondary metabolites
2.7.1.40	pyruvate kinase	ko01110	Biosynthesis of secondary metabolites
2.3.3.13	2-isopropylmalate synthase	ko01110	Biosynthesis of secondary metabolites
2.3.3.13	2-isopropylmalate synthase	ko01110	Biosynthesis of secondary metabolites
2.7.1.40	pyruvate kinase	ko01110	Biosynthesis of secondary metabolites
2.3.3.1	citrate synthase	ko01110	Biosynthesis of secondary metabolites
2.7.2.1	acetate kinase	ko01120	Microbial metabolism in diverse environments
2.7.1.40	pyruvate kinase	ko01120	Microbial metabolism in diverse environments
4.1.1.31	phosphoenolpyruvate carboxylase	ko01120	Microbial metabolism in diverse environments
2.7.1.40	pyruvate kinase	ko01120	Microbial metabolism in diverse environments
2.3.3.1	citrate synthase	ko01120	Microbial metabolism in diverse environments
2.7.1.71	shikimate kinase	ko01130	Biosynthesis of antibiotics
2.7.1.40	pyruvate kinase	ko01130	Biosynthesis of antibiotics
2.7.1.40	pyruvate kinase	ko01130	Biosynthesis of antibiotics
2.3.3.1	citrate synthase	ko01130	Biosynthesis of antibiotics

2.7.2.1	acetate kinase	ko01200	Carbon metabolism
2.7.1.40	pyruvate kinase	ko01200	Carbon metabolism
4.1.1.31	phosphoenolpyruvate carboxylase	ko01200	Carbon metabolism
2.7.1.40	pyruvate kinase	ko01200	Carbon metabolism
2.3.3.1	citrate synthase	ko01200	Carbon metabolism
2.3.3.13	2-isopropylmalate synthase	ko01210	2-Oxocarboxylic acid metabolism
2.3.3.13	2-isopropylmalate synthase	ko01210	2-Oxocarboxylic acid metabolism
2.3.3.13	2-isopropylmalate synthase	ko01210	2-Oxocarboxylic acid metabolism
2.3.3.1	citrate synthase	ko01210	2-Oxocarboxylic acid metabolism
2.7.1.71	shikimate kinase	ko01230	Biosynthesis of amino acids
2.3.3.13	2-isopropylmalate synthase	ko01230	Biosynthesis of amino acids
2.7.1.40	pyruvate kinase	ko01230	Biosynthesis of amino acids
2.3.3.13	2-isopropylmalate synthase	ko01230	Biosynthesis of amino acids
2.3.3.13	2-isopropylmalate synthase	ko01230	Biosynthesis of amino acids
2.7.1.40	pyruvate kinase	ko01230	Biosynthesis of amino acids
2.3.3.1	citrate synthase	ko01230	Biosynthesis of amino acids
2.7.1.40	pyruvate kinase	ko04922	Glucagon signaling pathway
2.7.1.40	pyruvate kinase	ko04922	Glucagon signaling pathway

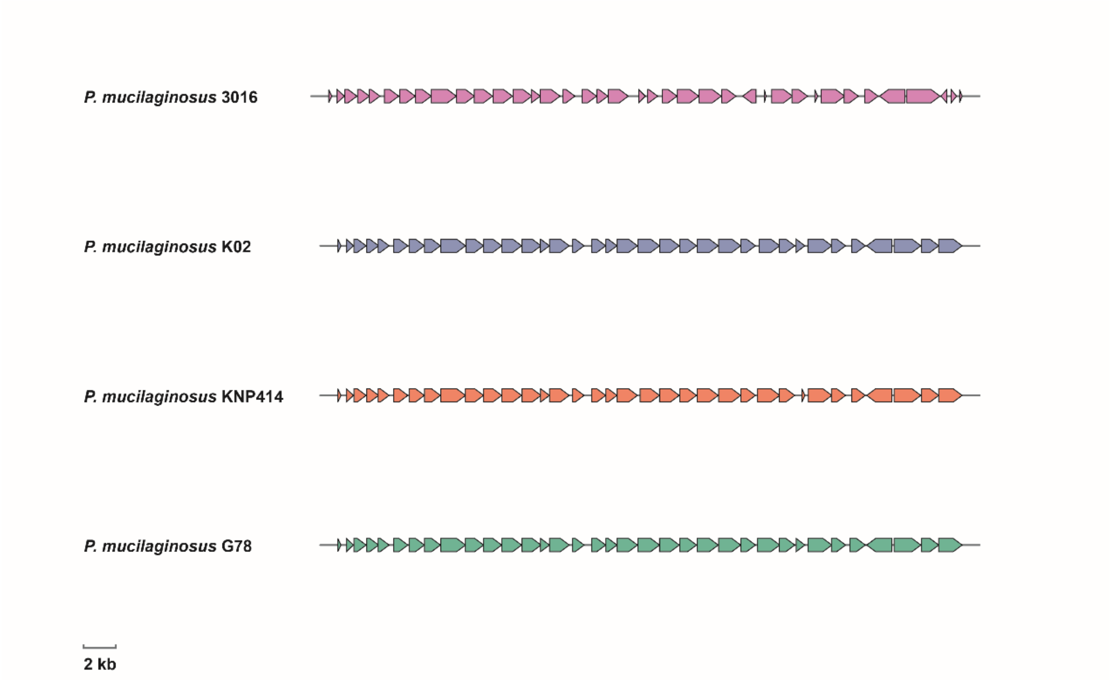

Figure S1. Predicted exopolysaccharide gene clusters for four *Paenibacillus mucilaginosus* strains (3016, K02, KNP414 and G78).

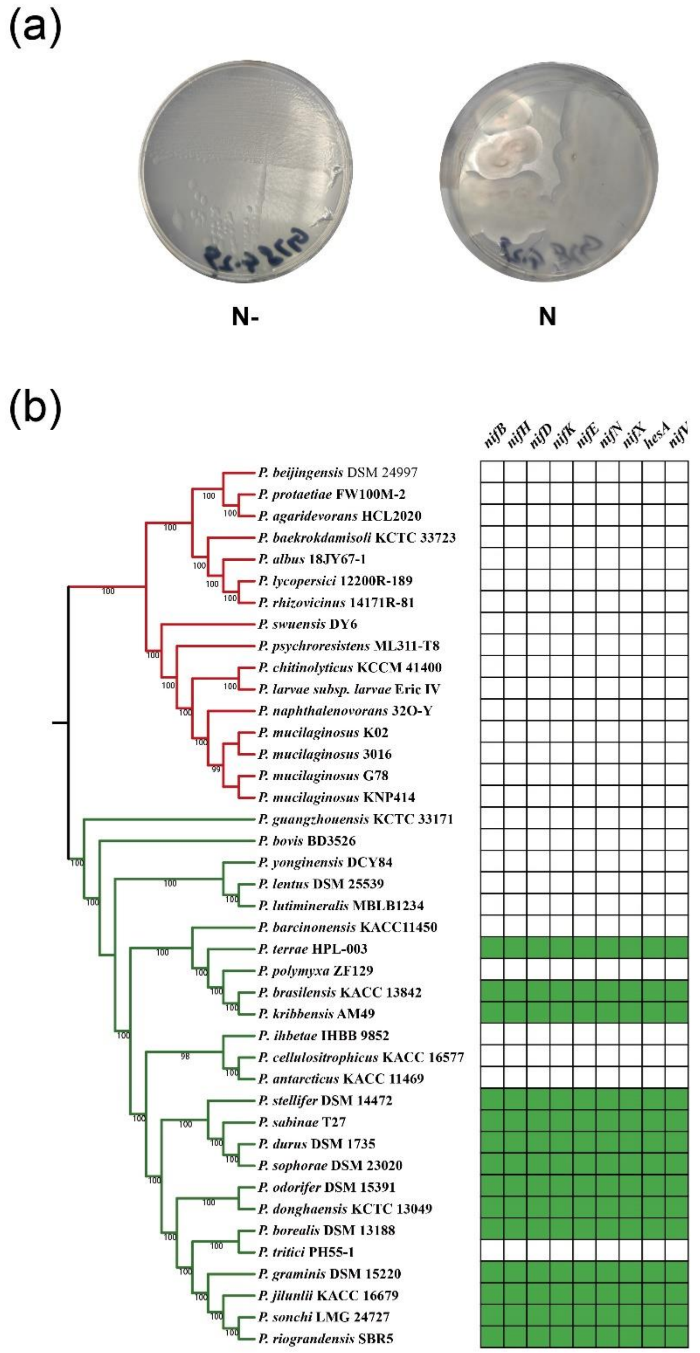

Figure S2. The growth ablity of *P. mucilaginosus G78* on nitrogen free medium and genes involved in nitrogen fixation of 41 *Paenibacillus* strains. (a) The colonies of *P. aenibacillus* G78 on the N agar medium with or without nitrogen. N-, the N medium without KNO_3; N, the N medium. (b) Genes involved in nitrogen fixation of 41 *Paenibacillus* strains. Colored box represents the presence of a gene within a genome and white box indicates absence of a gene.
